# METTL3 Promotes Esophageal Squamous Cell Carcinoma Metastasis Through Enhancing GLS2 Expression

**DOI:** 10.3389/fonc.2021.667451

**Published:** 2021-05-19

**Authors:** Xiaoting Chen, Lanlan Huang, Tingting Yang, Jiexuan Xu, Chengyong Zhang, Zhendong Deng, Xiaorong Yang, Naihua Liu, Size Chen, Shaoqiang Lin

**Affiliations:** ^1^ Clinical Department of Guangdong Metabolic Disease Research Centre of Integrated Chinese and Western Medicine, The First Affiliated Hospital of Guangdong Pharmaceutical University, Guangzhou, China; ^2^ School of Clinical Medicine, Guangdong Pharmaceutical University, Guangzhou, China; ^3^ Clinical Laboratory, The First Affiliated Hospital of Guangdong Pharmaceutical University, Guangzhou, China; ^4^ School of Traditional Chinese Medicine, Guangdong Pharmaceutical University, Guangzhou, China; ^5^ Guangdong Provincial Engineering Research Center for Esophageal Cancer Precise Therapy, The First Affiliated Hospital of Guangdong Pharmaceutical University, Guangzhou, China

**Keywords:** ESCC, METTL3, m6A, GLS2, metastasis

## Abstract

Recent studies have identified pleiotropic roles of methyltransferase-like 3 (METTL3) in tumor progression. However, the roles of METTL3 in esophageal squamous cell carcinoma (ESCC) are still unclear. Here, we investigated the function and mechanism of METTL3 in ESCC tumorigenesis. We reported that higher METTL3 expression was found in ESCC tissues and was markedly associated with depth of invasion and poor prognosis. Loss- and gain-of function studies showed that METTL3 promoted the migration and invasion of ESCC cells *in vitro*. Integrated methylated RNA immunoprecipitation sequencing (MeRIP-Seq) and RNA sequencing (RNA-Seq) analysis first demonstrated that glutaminase 2 (GLS2) was regulated by METTL3 *via* m6A modification. Our findings identified METTL3/GLS2 signaling as a potential therapeutic target in antimetastatic strategies against ESCC.

## Introduction

Esophageal cancer is one of the most common malignant tumors worldwide, and it is the third most common cancer in China (1, 2). For all stages combined, the 5-year relative survival rate for esophageal cancer is 20%, (localized 47%, regional 25%, distant 5%) (1). Metastasis is a major cause of death in esophageal cancer patients. Esophageal squamous cell carcinoma (ESCC) is the major histological subtype of esophageal cancer in China (3). Metastasis is found in over 60% of newly diagnosed cases, which is one of the principal reasons why the overall 5-year survival rate of esophageal cancer is <15% (4). Hence, identification of novel functional genes that contribute to metastasis and clarification of the underlying molecular mechanisms are essential for the development of effective therapeutic strategies.

Epigenetics has increasingly been recognized for its role in tumor formation and progression (5–8). Traditionally, epigenetics includes DNA methylation, histone modification, nucleosome repositioning and post-transcriptional gene regulation by miRNAs (5–9). Dysregulation of epigenetic modifying genes profoundly contributes to human diseases and has been frequently reported in multiple types of cancer (10–12), including ESCC (13, 14). Similarly, RNAs also carry hundreds of various sites for distinct post-transcriptional modifications, generating a new field known as “epitranscriptomics” (15). Among numerous RNA modifications, N6-methyladenosine (m6A) is the most abundant mRNA modification in eukaryotic cells (16, 17). Accumulating evidence suggests that m6A RNA methylation strongly impacts RNA metabolism and is involved in the pathogenesis of many kinds of diseases, including cancers (18–21). Similar to DNA methylation, m6A modification is reversible and catalyzed by corresponding enzymes, namely, “writers,” “erasers,” and “readers”. M6A writers, erasers and readers are proteins that can add, remove, or recognize m6A on mRNAs, respectively (22–24). Addition of m6A is catalyzed by a methyltransferase complex (MTC) composed of several proteins (25). Methyltransferase-like protein 3 (METTL3) is an S-adenosyl methionine (SAM)-binding protein; METTL3 is the most important component of the m6A MTC and is highly conserved in eukaryotes from yeast to humans (26). Dysregulation of METTL3 expression has been reported in colorectal cancer (27, 28), pancreatic cancer (18) and gastric cancer (19, 29). Several recent reports suggest that METTL3 promotes cancer cell metastasis (19, 27–30). METTL3 regulates colorectal cancer metastasis by enhancing the mRNA stability of SOX2, HK2 and SLC2A1 (GLUT1) through an m6A-IGF2BP2/3-dependent mechanism (27, 28). METTL3 promotes mRNA methylation, enhances the stability of HDGF and ZMYM1 and promotes gastric cancer metastasis (19, 29). METTL3 has been shown to induce epithelial-mesenchymal transition (EMT), the early event of metastasis by YTHDF1 mediating m6A-increased translation of Snail mRNA (30). The expression of METTL3 was shown to be significantly increased in ESCC tissues (31). However, the functional roles and the underlying mechanisms of METTL3 in ESCC remain largely unknown.

Glutamine (Gln) is the most abundant amino acid in human plasma (32). Many cancers exhibit a notable preference for Gln in respiration (33). Humans have two genes encoding glutaminase enzymes, glutaminase 1 (GLS1) and GLS2. GLS1 has been shown to be associated with Gln addiction in tumors and has oncogenic properties (34–36), whereas the function of GLS2 in cancer is less well defined and appears to be context dependent (37–45). GLS2 has been reported as a tumor suppressor in some types of cancer (37, 43–45). GLS2 repressed cell migration, invasion and metastasis of in hepatocellular carcinoma (37) and induced growth inhibition in glioma cell lines (45). However, increased expression of GLS2 promoted metastasis and increased mortality risk in breast cancer (41).

Here, we show that the expression of METTL3, a major RNA N6-adenosine methyltransferase, was upregulated in ESCC. Clinically, elevated METTL3 levels were predictive of poor prognosis. Functionally, we found that METTL3 promoted the migration and invasion of ESCC cells *in vitro*. Mechanistically, we unveiled the METTL3-mediated m6A modification profile in ESCC cells for the first time and identified GLS2 as a downstream target of METTL3. Accordingly, GLS2 knockdown decreased the cell migration and invasion. In conclusion, we identified METTL3/GLS2 signaling as a potential therapeutic target in antimetastatic strategies against ESCC.

## Materials and Methods

### Patients and Tissue Specimens

ESCC tissue microarray (TMA) slides were purchased from Shanghai Outdo Biotech Co., Ltd., and were collected between 2009 and 2015. The ESCC TMA slide (HEsoS180Su07) contains 101 tumor tissues and 53 adjacent normal tissues. All samples were confirmed by pathological examination according to the American Joint Committee on Cancer (AJCC) Cancer Staging Manual (8th edition). The clinical characteristics of all patients are listed in **Table S1**. Written informed consent for the biological studies was obtained from the patients or their guardians. All experiments were approved by the Ethics Committee of The First Affiliated Hospital of Guangdong Pharmaceutical University.

### Immunohistochemistry (IHC)

IHC staining was performed as described previously (46). Briefly, TMAs were treated with xylene and 100% ethanol, followed by decreasing concentrations of ethanol. After antigen retrieval, TMAs were blocked and stained with anti-METTL3 antibody (1:500, Abcam, USA), followed by incubation with secondary antibody and standard avidin biotinylated peroxidase complex. Hematoxylin was used for counterstaining, and images were obtained with an Image acquisition system (Olympus, Japan). The total METTL3 immunostaining score was calculated as the sum of the score for the proportion of positively stained tumor cells (PP) and the score for staining intensity (SI). PP was scored with a four-point scale: 0 (< 5%), 1 (5–25%), 2 (25–50%), 3 (50%-75%), and 4 (>75%), and SI was scored on a scale of 0 to 3 (0, negative staining; 1, weak staining; 2, moderate staining; and 3, strong staining). The final staining score was calculated by multiplying the SI and PP scores, resulting in a score value ranging from 0 to 12. The median value of the total staining score was 4; thus, a score of 0-4 was defined as low expression, and a score of 4-12 was defined as high expression.

### Cell Culture and Transfection

The human esophageal epithelial cell line HEEC and the ESCC cell lines TE1, TE13, Eca109 and EC-1 were obtained from the Beina Chuanglian Biotechnology Research Institute (Beijing, China). All cells were cultured in Dulbecco’s modified Eagle’s medium (DMEM) supplemented with 10% fetal bovine serum and antibiotics (100 μg/mL penicillin and 100 μg/mL streptomycin) at 37°C in a humidified incubator with 5% CO_2_.

### Lentiviral Packaging and Cell Transduction

METTL3 knockdown or overexpression lentiviruses were obtained from Shanghai GeneChem Co., Ltd, China. For overexpression, cDNA was amplified by PCR and subcloned into the GV341 vector according to the manufacturer’s instructions. For stable silencing, shRNA lentiviruses (sh-METTL3 with target sequence GCCTTAACATTGCCCACTGAT) were constructed using GV248 vectors. TE13 and TE1 cells were plated in 24-well dishes at 20-30% confluence and infected with METTL3 overexpression lentivirus (LV-METTL3), negative control (LV-NC), METTL3 knockdown lentivirus (sh-METTL3), or a scramble control (sh-NC). Pools of stable transduced cells were generated by selection using puromycin (1 μg/ml) for 2 weeks. METTL3 and GLS2 siRNAs were ordered from RiboBio Co., Ltd, China (METTL3 with target sequence CAAGTATGTTCACTATGAA; GLS2_1 with target sequence CGGCTATTATCTCAAGGAA; GLS2_2 with target sequence GGTCAATGCTGGTGCCATT). Transfection was achieved by using Lipofectamine 3000 (Invitrogen) following the manufacturer’s protocols. After transfection, the expression of GLS2 was validated by qRT-PCR analysis and Western blots.

### RNA Extraction and Quantitative Real-Time PCR (qRT-PCR)

Total RNA was extracted from cells by using RNAiso Plus (TaKaRa, Japan) according to the manufacturer’s protocol. cDNA was synthesized using a TransScript One-Step gDNA Removal and cDNA Synthesis SuperMix kit (Transgen, China) and qRT-PCR for mRNA was performed using an UltraSYBR Mixture kit (ComWin Biotech, China). The relative gene expression of mRNAs was calculated by using the 2-ΔΔCt method. Glyceraldehyde 3-phosphate dehydrogenase (GAPDH) was used as the endogenous standard control for mRNA detection. Each sample was assayed in triplicate, and data were analyzed by comparing Ct values. All PCR primers were purchased from Igebiotech (Guangzhou, China). All primers used in this study are as follows: *METTL3_*F TTGTCTCCAACCTTCCGTAGT and *METTL3_*R: CCAGATCAGAGAGGTGGTGTAG; *GLS2*_F: CTGCACTAAAGGCCACTGGA and *GLS2*_R: TCTTTCGGAATGCCTGGGTC; *GAPDH*_F: AGCCTCAAGATCATCAGC and *GAPDH*_R: GAGTCCTTCCACGATACC.

### Western Blotting (WB) Analysis

Total cellular proteins were lysed by RIPA buffer containing protease inhibitors (Beyotime, China). Protein extracts were harvested and quantified by bicinchoninic acid analysis (Beyotime, China). Protein extracts were separated by 10% SDS-PAGE and transferred onto polyvinylidene fluoride (PVDF) membranes (Bio-Rad, USA). After incubation with primary antibodies, the membranes were then incubated with peroxidase (HRP)-conjugated secondary antibody (1:3000, Beyotime, China). After washing, signals were detected using a chemiluminescence system (Sagecreation, China). Anti-METTL3 (1:1000 dilution, Abcam, Cambridge, UK), anti-GLS2 (1:1000, Sigma-Aldrich, St-Louis, MO, USA), anti-β-actin (1:1000, Cell Signaling Technology, USA) and anti-GAPDH (1:1000 dilution, Cell Signaling Technology, USA) antibodies were used.

### Wound Healing Assay

TE13 cells (6.5×10^4^) or TE1 cells (4.5×10^4^) were seeded in each well of a Culture-Insert 2 Well (ibidi, Germany), which was placed in a 24-well plate. After incubation for 24 h, a scratch was made after removing the Culture-Insert 2 Well by using sterile tweezers. The plate was washed gently twice and cultured in DMEM supplemented with 1% FBS at 37°C with 5% CO_2_. The results of cell migration were recorded at 0, 6 and 9 h by a microscope. Each assay was repeated three times.

### Cell Migration Assay

Cell migration assays were carried out by transwell chamber assays (Corning, US). TE13 cells (1×10^5^) or TE1 cells (5×10^4^) were seeded in the upper chamber in serum-free medium, and the lower side was filled with DMEM containing 10% FBS. After incubation at 37°C in 5% CO_2_ for a suitable time (TE13 cells: 27 h, TE1 cells: 12 h), the upper chambers were fixed with 4% paraformaldehyde for 15 minutes, stained with 0.1% crystal violet and photographed using a microscope. All studies were repeated at least three times in triplicate.

### Cell Invasion Assay

Transwell chambers precoated with Matrigel (Corning, US) were used to perform the invasion assay. A total of 1×10^5^ TE13 cells or 5×10^4^ TE1 cells were seeded in the upper chamber in serum-free medium, and the lower side was filled with DMEM containing 10% FBS. After incubation at 37°C in 5% CO_2_ for a suitable time (TE13 cells: 30 h, TE1 cells: 15 h), the upper chambers were fixed with 4% paraformaldehyde for 15 minutes, stained with 0.1% crystal violet and photographed using a microscope. All studies were repeated at least three times in triplicate.

### Methylated RNA Immunoprecipitation Sequencing (MeRIP-Seq)

Total RNA from the transfected TE13 cells was extracted using RNAiso Plus (TaKaRa, Japan) following the manufacturer’s procedure. The total RNA quality and quantity were analyzed by a Bioanalyzer 2100 and RNA 6000 Nano LabChip Kit (Agilent, CA, USA) with RIN number >7.0. Approximately 200 µg of total RNA was subjected to isolation of poly (A) mRNA with poly-T oligo-attached magnetic beads (Invitrogen). Following purification, the poly(A) mRNA fractions are fragmented into ~100-nt-long oligonucleotides using divalent cations under elevated temperature. Then, the cleaved RNA fragments were incubated for 2 h at 4°C with m6A-specific antibody (No. 202003, Synaptic Systems, Germany) in IP buffer (50 mM Tris-HCl, 750 mM NaCl and 0.5% Igepal CA-630) supplemented with BSA (0.5 μg μl−1). The mixture was then incubated with protein-A beads and eluted with elution buffer (1 × IP buffer and 6.7 mM m6A). Eluted RNA was precipitated by 75% ethanol. Eluted m6A-containing fragments (IP) and untreated input control fragments were converted to the final cDNA library in accordance with strand-specific library preparation by the dUTP method. The average insert size for the paired-end libraries was ~100 ± 50 bp. Then, we performed the paired-end 2×150 bp sequencing on an Illumina NovaSeq™ 6000 platform at LC-Bio Biotech, Ltd., (Hangzhou, China) following the vendor’s recommended protocol.

### RNA-Binding Protein Immunoprecipitation (RIP) Assay

RNA immunoprecipitation was performed using the Magna RIP RNA-Binding Protein Immunoprecipitation Kit (Millipore) following the manufacturer’s protocol. Briefly, after formaldehyde -crosslinking (0.3% for 10 min), 2 × 10^7^ TE13 cells were lysed in RIP lysis buffer. Magnetic Bead Protein A/G was incubated with 5 μg of an anti-METTL3 rabbit antibody (ab195352, Abcam) or normal rabbit IgG for 1 hour at room temperature. Then, the coated beads were incubated with prepared cell lysates overnight at 4°C. Then, the RNA was extracted and resuspended in 10 μL of RNase-free water. The RNAs were analyzed by qRT-PCR.

### Statistical Analysis

The statistical analysis was performed using SPSS 18.0 software (SPSS, IBM, Chicago, IL, USA). The data are expressed as the mean ± SD, and statistical significance was determined with Student’s *t* test. Statistical comparisons between groups were performed using Student’s paired *t* test. *P* values less than 0.05 were considered statistically significant. The association between METTL3 staining and clinicopathologic parameters was evaluated with the chi-square test. The cumulative survival time was calculated utilizing the Kaplan-Meier method and analyzed with the log-rank test. Univariate and multivariate analyses were performed based on the Cox proportional hazards regression model.

## Results

### METTL3 Is Highly Expressed in ESCC

**Figure 1:**
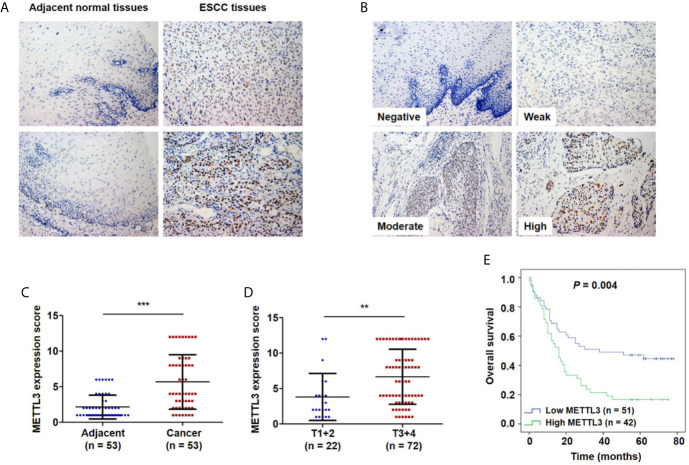
Upregulation of METTL3 expression in ESCC tissues is strongly associated with poor prognosis. **(A)** Representative immunostaining of METTL3 in sections from esophageal tumors and adjacent normal tissues. Photomicrographs obtained at × 200 magnification. **(B)** Representative METTL3 expression in ESCC with negative, weak, moderate and high staining. **(C)** The IHC score of METTL3 in ESCC tissues was higher than that of adjacent normal tissues. Statistical significance was determined by a two-tailed, paired Student’s *t* test. **(D)** METTL3 expression was significantly associated with depth of invasion. Statistical significance was determined using a two-tailed, unpaired Student’s *t* test. **(E)** Kaplan–Meier analysis of overall survival according to low and high METTL3 protein expression in 93 ESCC patients. (**P < 0.01, ***P < 0.001).

To investigate the clinical significance of METTL3 expression in ESCC, we used IHC to examine METTL3 expression in 101 tumor tissues and 53 adjacent normal tissues. METTL3 staining was localized in the nucleus of cells (**Figures 1A, B**). As shown in **Figure 1A**, adjacent normal squamous epithelia had only a few scattered cells with high METTL3 expression in the basal layer. In contrast, strong METTL3 staining was observed in ESCC tumor tissues. Representative photos of ESCC cells with different METTL3 expression intensities were shown in **Figure 1B**. The IHC results showed that METTL3 expression was significantly higher in 71.7% (38/53) of the ESCC tissues than in the adjacent normal tissues (**Figure 1C**). We further examined METTL3 expression in four human ESCC cell lines (Eca109, TE1, TE13 and EC-1) and a normal esophageal epithelial cell line (HEEC). We also found that METTL3 expression was higher in the ESCC cell lines than in the HEEC cell line (**Figures S1A, B**). Moreover, METTL3 expression was found to be significantly upregulated in other types of cancer, such as hepatocellular carcinoma (HCC) and cholangiocarcinoma (**Figure S1C**).

### The Expression of METTL3 Was Correlated With the Depth of Invasion and Poor Prognosis

We further investigated the relationship of METTL3 expression with the clinicopathological features. The median expression value of METTL3 was used as a cut-off point to separate the patients into high and low expression groups. As shown in **Table 1**, METTL3 expression was significantly associated with depth of invasion (*P* = 0.007). There was no significant association between METTL3 expression and age (*P* = 0.373), sex (*P* = 0.352), lymph node metastasis (*P* = 0.927) or TNM stage (*P* = 0.850). METTL3 was significantly upregulated in ESCC with more advanced T grades (**Figure 1D**; *P* < 0.0001). Kaplan–Meier analysis showed that high levels of METTL3 expression were correlated with poor overall survival in ESCC (**Figure 1E**). Univariate analysis showed that overall survival was correlated with METTL3 expression (**Table 2**). Further, multivariate Cox regression analysis revealed that METTL3 expression was an independent prognostic factor for poor survival (**Table 3**). Additionally, by using Gene Expression Profiling Interactive Analysis (GEPIA) we found that high expression of METTL3 was correlated with poor survival in patients with HCC (**Figure S1D**).

**Table 1:** Correlation between METTL3 expression and clinicopathologic features of ESCC patients (n = 101).

Factors	Number of cases	METTL3 expression		*P* value
		Low	High
**Age**				0.427
<65	50	25 (45.5%)	25 (54.3%)	
≥65	51	30 (54.5%)	21 (45.7%)	
**Gender**				0.429
Female	84	44 (80.0%)	40 (87.0%)	
Male	17	11 (20.0%)	6 (13.0%)	
**pT**				0.008
T1+T2	22	17 (34.7%)	5 (11.1%)	
T3+T4	72	32 (63.5%)	40 (88.9%)	
**pN**				1
N0	51	28 (50.9%)	23 (50.0%)	
N+	50	27 (49.1%)	23 (50.0%)	
**TNM stage**				1
I+II	49	26 (53.1%)	23 (51.1%)	
III	45	23 (46.9%)	22 (48.9%)	

**Table 2:** Overall survival of ESCC patients: univariate analysis.

Factors	HR	95% CI	*P* value
Gender	0.310	0.092-1.049	0.060
Age	1.972	0.745-5.223	0.172
pT	1.503	0.490-4.605	0.476
pN	2.134	0.837-5.440	0.112
TNM stage	2.134	0.837-5.440	0.112
METTL3	2.718	1.048-7.047	0.040^*^

*P < 0.05; HR, hazard ratio; CI, confidence interval.

**Table 3:** Overall survival of ESCC patients: multivariate analysis.

Factors	HR	95% CI	*P* value
Gender	0.398	0.178-0.888	0.024
pN	1.789	1.062-3.015	0.029
METTL3	1.919	1.134-3.247	0.015

HR, hazard ratio; CI, confidence interval.

### Silencing METTL3 Expression Inhibits the Migration and Invasion of ESCC Cells

**Figure 2:**
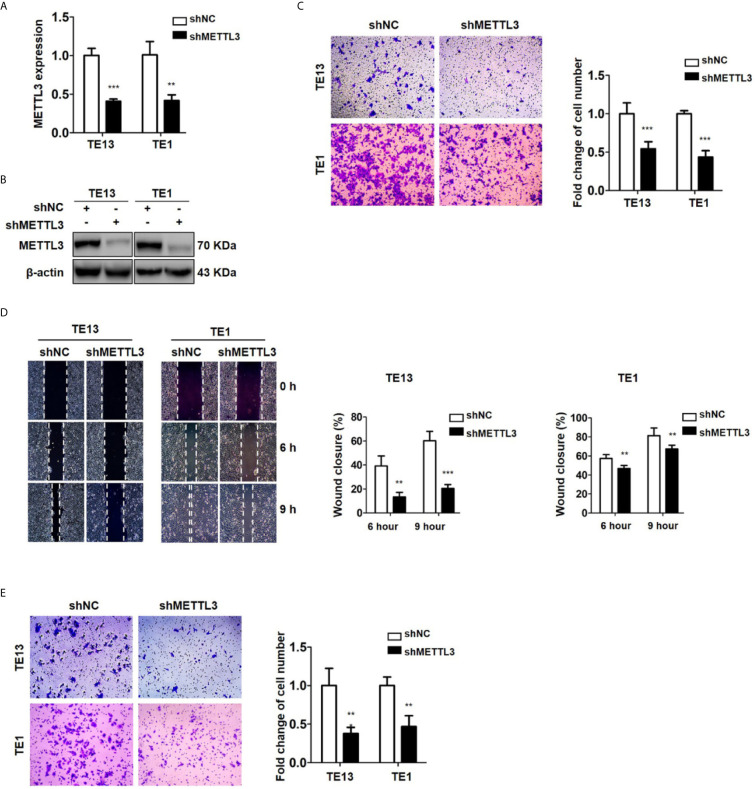
Downregulation of METTL3 expression inhibits the migration and invasion of ESCC cell lines. **(A)** qRT-PCR analysis of the expression of METTL3 in ESCC cells infected with pLenti-shMETTL3 or pLenti-vector. **(B)** Western blot analyses of the expression of METTL3 extracts from ESCC cells infected with pLenti-shMETTL3 or pLenti-vector. **(C)** Transwell migration assays were used to estimate the effects of downregulation of METTL3 on ESCC cell migration. **(D)** Wound healing assays were used to detect cell migration in ESCC cells after METTL3 shRNA transfection. **(E)** Transwell invasion assays were used to estimate the effects of downregulation of METTL3 expression on ESCC cell invasion. (The quantitative data are presented in the histograms and were assessed with a two-tailed unpaired Student’s *t* test. Data are presented as the mean ± SD. **P < 0.01, ***P < 0.001).

METTL3 expression was found to be upregulated in lung cancer, breast cancer, liver cancer and glioblastoma (47–50) and associated with metastasis in lung cancer (47) and oral squamous cell carcinoma (51). To explore the role of METTL3 in ESCC, we used pLenti-shMETTL3 and siRNA to knockdown endogenous METTL3 expression in ESCC cells. Our results showed that METTL3 shRNA and siRNA transfection significantly reduced the expression of METTL3 in ESCC cell lines (**Figures 2A, B** and **S2A**). Cell migration was determined by transwell migration and wound healing assays. We found that downregulation of METTL3 expression inhibited the migration of TE13 and TE1 cells (**Figures 2C**; **S2B**). Wound healing assays showed that downregulation of METTL3 expression led to a decrease in cell wound healing in TE13 and TE1 cells (**Figure 2D**). Transwell invasion assays were conducted to measure cell invasion in ESCC cells after METTL3 knockdown. The results shown that downregulation of METTL3 expression substantially suppressed the invasion of ESCC cells (**Figures 2E** and **S2C**). This finding suggested that METTL3 knockdown could effectively inhibit the migration and invasion of ESCC cells.

### Overexpression of METTL3 Promotes Cell Migration and Invasion

**Figure 3:**
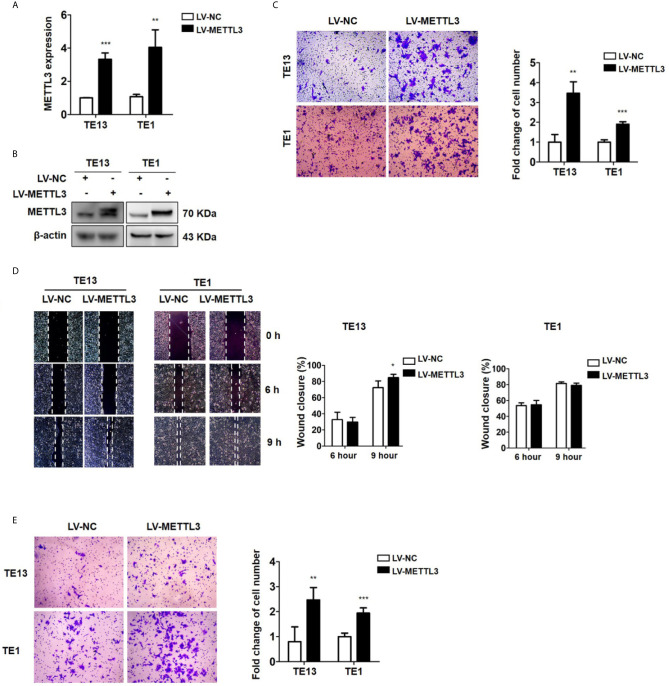
Overexpression of METTL3 promotes cell migration and invasion. **(A)** qRT-PCR analysis of the expression of METTL3 in ESCC cells infected with pLenti-METTL3 or pLenti-vector. **(B)** Western blot analyses of the expression of METTL3 extracts from ESCC cells infected with pLenti-METTL3 or pLenti-vector. **(C)** Transwell migration assays revealed that overexpression of METTL3 increased the migration of TE13 and TE1 cells. **(D)** Cell migration was assessed in wound healing assays. **(E)** Transwell invasion assays revealed that overexpression of METTL3 increased the invasion of TE13 and TE1 cells. (The quantitative data are presented in the histograms and were assessed with a two-tailed unpaired Student’s *t* test. Data are presented as the mean ± SD. *P < 0.05, **P < 0.01, ***P < 0.001).

To detect the effect of METTL3 on ESCC, we then transfected ESCC cells with METTL3 lentiviral vectors. The expression of METTL3 was increased, as shown by qRT-PCR and Western blots (**Figures 3A, B** and **S3A**). Transwell migration assays showed that METTL3 overexpression substantially increased the migration of ESCC cells (**Figures 3C** and **S3B**). Wound healing assays showed that METTL3 overexpression substantially accelerated wound closure in TE13 cells (**Figure 3D**). In addition, transwell invasion assays showed that the number of invading cells was significantly higher in the METTL3-overexpressing cells than in the control cells (**Figure 3E**). Our findings indicated that METTL3 overexpression increased cell migration and invasion in ESCC cells.

### MeRIP-Seq Analysis Reveals the m6A Profiles in ESCC Cells

**Figure 4:**
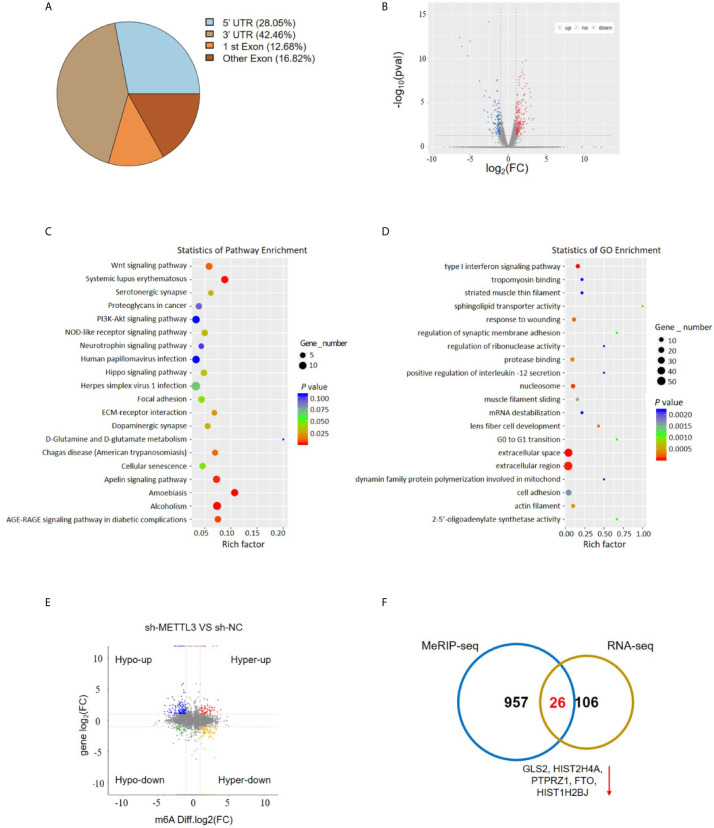
MeRIP-Seq analysis unveils the m6A profiles in the ESCC cells. **(A)** Proportion of m6A peak distribution in the 5’UTR, start codon, 3’UTR, first exon or other exon region across the entire set of mRNA transcripts. **(B)** The volcano plot for RNA-Seq analysis showed the differential expression of transcripts, including upregulated and downregulated transcripts. **(C)** Pathway analysis showed the potential pathway for ESCC. **(D)** Gene Ontology analysis showed the target mechanisms for ESCC. **(E, F)** The shared genes between MeRIP-Seq and RNA-seq.

To investigate the downstream target mRNAs of METTL3, we performed m6A-RNA immunoprecipitation sequencing (MeRIP-Seq) to compare the global profiling of m6A target genes between the control and METTL3 stable knockdown cells. The MeRIP-Seq results showed that a proportion of m6A peak distributions displayed m6A peaks in the 3’ untranslated region (UTR), 5’ UTR, exons and coding sequences (CDSs) (**Figure 4A**). In total, 1111 and 802 m6A peaks presented significant decrease and increase, respectively, in the METTL3 knockdown cells relative to the control cells (|log_2_(FC)| ≥0.5, *P*<0.05; **Supplementary Material**). Since METTL3 mediates m6A modification, the 1111 decreased peaks are theoretically anticipated to include genuine targets of METTL3. Therefore, the association between these peaks and differentially expressed genes was identified by RNA-seq. A total of 132 genes were differentially expressed by at least 2-fold in the METTL3 knockdown cells (**Figure 4B**). Pathway analysis showed the potential pathway for ESCC (**Figure 4C**). Gene Ontology (GO) analysis showed the target mechanisms for ESCC (**Figure 4D**). We then integrated the RNA-Seq analysis with the MeRIP-Seq analysis. Filtering the 1111 decreased m6A peaks (harbored by 983 genes) within the 132 differentially expressed genes resulted in the identification of 32 peaks harbored by 26 genes (5 of them were downregulated), suggesting that knockdown of METTL3 expression might reduce the m6A levels of these 26 gene transcripts and thus result in the altered expression of these transcripts (**Figures 4E, F** and **Table S2**).

### GLS2 as a Downstream Target of METTL3

**Figure 5:**
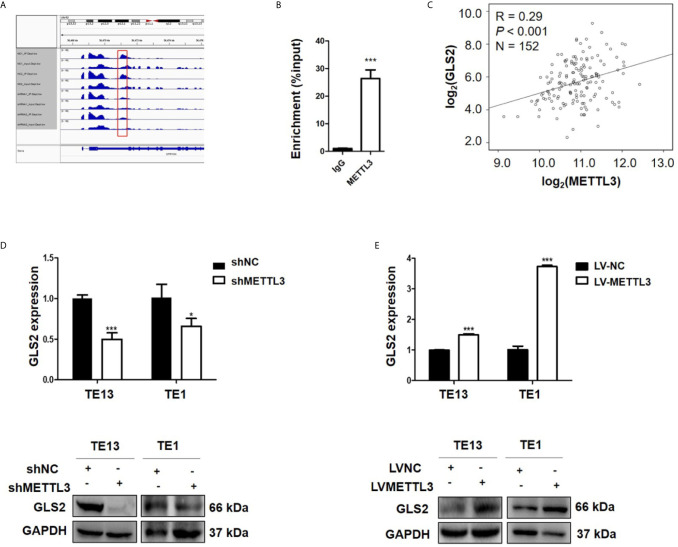
GLS2 as a downstream target of METTL3. **(A)** m6A peaks were decreased in 3’UTRs of GLS2 genes in the METTL3 knockdown cells. **(B)** RIP**-**qRT-PCR showing the binding of METTL3 to GLS2. **(C)** TCGA database illustrated the correlation between GLS2 and METTL3 expression. **(D)** GLS2 expression in TE13 and TE1 cells with stable METTL3 knockdown by using qRT-PCR and Western blots. **(E)** qRT-PCR and Western blot analysis of GLS2 expression in TE13 and TE1 cells with stable METTL3 overexpression. Data are presented as the mean ± SD. *P < 0.05, ***P < 0.001.

**Figure 6:**
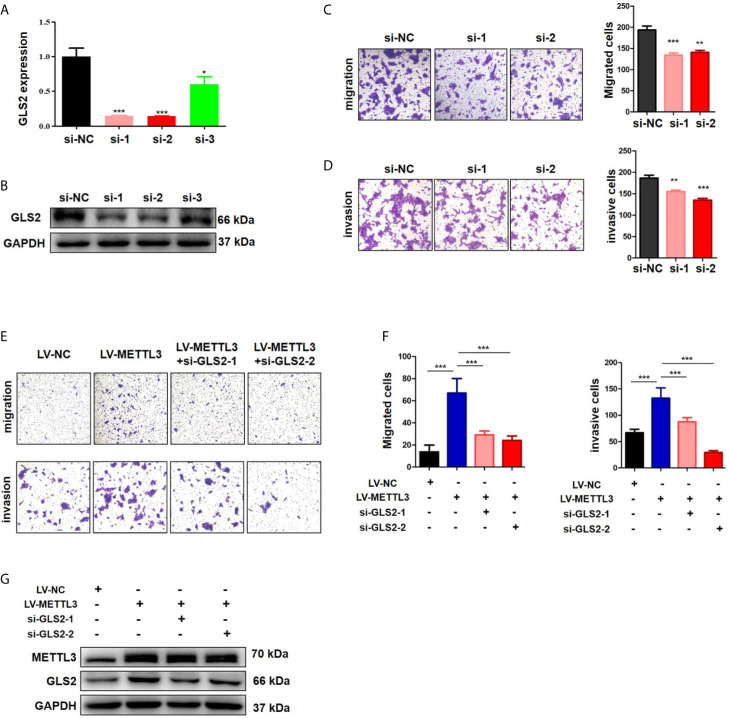
METTL3 promoted ESCC cells migration and invasion in a GLS2-dependent manner. **(A)** qRT-PCR analysis of GLS2 expression in TE13 cells with transient GLS2 knockdown. **(B)** Western blot analysis of GLS2 expression in TE13 cells with transient GLS2 knockdown. **(C)** Transwell migration assays were used to estimate the effects of GLS2 downregulation on TE13 cells migration. **(D)** Transwell invasion assays were used to estimate the effects of downregulation of GLS2 on TE13 cells invasion. **(E, F)** Transwell assays were used to detect the effects of downregulation of GLS2 on METTL3-overexpression cells migration and invasion. A total of 8×10^4^ cells were seeded in the upper chamber in serum-free medium for a suitable time (migration: 24 h, invasion: 35 h), then the migrated cells were fixed and stained. **(G)** Western blot analysis of METTL3 and GLS2 expression. Data are presented as the mean ± SD. *P < 0.05, **P < 0.01, ***P < 0.001.

METTL3 could enhance target transcripts stability in a YTHDF1/IGF2BP2-mediated m6A-dependent manner (27, 30, 51, 52); therefore, we focused on the candidate genes with downregulated expression. Among these 5 genes with downregulated expression, m6A peaks in the UTRs of GLS2 showed a significant decrease in the METTL3 knockdown cells, and its expression demonstrated a significant shift after METLL3 knockdown (**Figure 5A**, **Table S3**). Next, RIP-qRT-PCR demonstrated strong binding of METTL3 with GLS2 in TE13 cells (**Figure 5B**). We also found that GLS2 expression was higher in ESCC cell lines than in HEEC cells (**Figure S4**), and GLS2 expression was strongly correlated with METTL3 expression (**Figure 5C**). Subsequently, we measured the RNA and protein expression of GLS2 upon silencing of METTL3. METTL3 knockdown resulted in significant downregulation of GLS2 expression at the RNA and protein levels (**Figures 5D**; **S2A**). Conversely, METTL3 overexpression upregulated GLS2 expression at the RNA and protein levels (**Figure 5E**). To investigate the biological role of GLS2 in ESCC cell migration and invasion, we knocked down GLS2 in TE13 cells by transfection of siRNA. Our results showed that siRNA transfection significantly reduced the expression of GLS2 at the RNA and protein levels in ESCC cells (**Figures 6A, B**). Transwell migration assays showed that knockdown of GLS2 expression significantly repressed cell migration compared to that of the control groups (**Figure 6C**). Transwell invasion assays were conducted to measure cell invasion in ESCC cells after GLS2 siRNA transfection. As shown in **Figure 6D**, downregulation of GLS2 notably suppressed the invasion of ESCC cells. To better understand roles of GLS2 in the METTL3-mediated cell migration and invasion, we knocked down GLS2 in TE13 cells with stable METTL3 overexpression. Transwell assays showed that downregulation of GLS2 attenuated METTL3-mediated migration and invasion (**Figures 6E–G**). These results strongly suggested that METTL3 promoted the ESCC migration and invasion, at least partially, in a GLS2-dependent manner. Overall, these findings indicated that GLS2 is a downstream target of METTL3.

## Discussion

Accumulating evidence suggests that m6A RNA methylation substantially impacts RNA metabolism and is involved in the pathogenesis of many kinds of diseases, including cancer (53). However, the regulation of m6A modification in ESCC is still unclear. METTL3, a major catalytic enzyme in the m6A methyltransferase system, is dysregulated and plays a dual role (oncogene or tumor suppressor) in different human cancers (48, 49, 54, 55). METTL3 is implicated in many aspects of tumor progression, including tumorigenesis, proliferation, invasion, migration, cell cycle, differentiation, and viability (48, 49, 55).

In our research, we found that METTL3 expression was significantly upregulated in the ESCC tissue and cell lines. In addition, this ectopic overexpression was correlated with poor prognosis. These findings are consistent with the results from the work by Xia et al. (31). *In vitro* cellular experiments, and gain- and loss-of function assays, demonstrated that METTL3 could promote ESCC migration and invasion, indicating that METTL3 might act as an oncogene in ESCC tumorigenesis.

METTL3 could generate RNA methylation on the N6 nitrogen of adenosine. Because m6A modification relies on reader proteins to exert its biological functions, RNA transcripts might be sorted into different groups based on their readers. For example, METTL3 has been shown to promote Snail mRNA translation in a YTHDF1 mediated m6A-dependent manner (30). In HCC, METTL3 interacts with SOCS2 and induces the m6A on SOCS2 mRNA, thus repressing SOCS2 expression through a YTHDF2-dependent mechanism (49). In colorectal cancer, METTL3 can add m6A on the CDS regions of SOX2 transcripts to prevent SOX2 mRNA degradation *via* IGF2BP2 (27). METTL3 stabilizes HK2 and SLC2A1 expression through an m6A-IGF2BP2/3-dependent mechanism (28).

To investigate the mechanism of METTL3 in ESCC metastasis, we used MeRIP-Seq and RNA-Seq to identify the targets of METTL3. MeRIP-seq showed that the m6A peaks were significantly enriched in the region surrounding the stop codon, including the CDS and 3’UTR. We integrated the RNA-Seq analysis with the MeRIP-Seq analysis and identified 26 candidate genes. The expression levels of five of the 26 candidate genes (PTPRZ1, GLS2, HIST2H4A, FTO, HIST1H2BJ) were significantly downregulated after METLL3 knockdown, suggesting that METTL3-mediated m6A modification might regulate the transcription of these five genes. Then, we used a RIP assay to assess the direct interaction between GLS2 and METTL3, and the results demonstrated strong binding of METTL3 with GLS2 in TE13 cells. In functional cellular experiments, we found that METTL3 knockdown or overexpression strongly regulated GLS2 expression. Moreover, downregulation of GLS2 attenuated METTL3-mediated migration and invasion. Overall, these findings indicated that GLS2 is a downstream target of METTL3.

The glutaminase isoenzymes GLS1 and GLS2 are key enzymes for glutamine metabolism. Efforts to target glutamine catabolism for cancer therapy have focused on inhibiting GLS1, which is highly expressed and oncosupportive in diverse malignancies (56). The importance of GLS2 in tumorigenesis is still unclear. GLS2 has been described as a tumor suppressor factor in HCC (37, 43, 44, 57) and glioblastoma (45). However, GLS2 has been reported as an oncogene in breast cancer (40, 41) and cervical cancer (58). In breast cancer, GLS2 was linked to enhanced *in vitro* cell migration and invasion and *in vivo* lung metastasis (41). Marilia et al. reported that GLS2 can enhance the EMT markers vimentin and actin stress fibers in breast cancer cells, and ERK inhibition affected the proliferation and migration induced by GLS2, indicating that GLS2 can regulate migration and invasion through the ERK-ZEB1-vimentin axis. We report here that downregulation of GLS2 expression suppressed the migration and invasion of ESCC cells, indicating that GLS2 might act as an oncogene in ESCC.

In conclusion, our findings confirmed the oncogenic role of METTL3 in ESCC. We herein identified GLS2 as a downstream target of METTL3. Our findings uncover METTL3/GLS2 signaling as a potential therapeutic target in antimetastatic strategies against ESCC.

## Data Availability Statement

The datasets presented in this study can be found in online repositories. The names of the repository/repositories and accession number(s) can be found in the article/**Supplementary Material**.

## Ethics Statement

The studies involving human participants were reviewed and approved by The First Affiliated Hospital of Guangdong Pharmaceutical University. The patients/participants provided their written informed consent to participate in this study.

## Author Contributions

SL and SC designed and supervised the study. XC and LH performed the experiments, analyzed the data, and wrote the paper. TY, JX, CZ, and ZD performed the experiments and analyzed the data. XY and NL corrected the manuscript. All authors contributed to the article and approved the submitted version.

## Funding

The present study was supported by the National Natural Science Foundation of China (81071751, 81802953), Guangzhou Science and Technology Project (201704030059), Science and Technology Planning Project of Guangdong Province of China (2020A0505100058) and Special Innovative Projects of Guangdong Province (2019KZDXM024).

## Conflict of Interest

The authors declare that the research was conducted in the absence of any commercial or financial relationships that could be construed as a potential conflict of interest.
