# Supplementary material for: METTL3 Promotes Esophageal Squamous Cell Carcinoma Metastasis Through Enhancing GLS2 Expression
Source: Front Oncol. 2021 May 19;11:667451. doi: 10.3389/fonc.2021.667451 (PMC8170325; doi:10.3389/fonc.2021.667451)
Supplement: Supplementary file 2 [file DataSheet_2.docx]

Supplementary Material

## Supplementary Figures


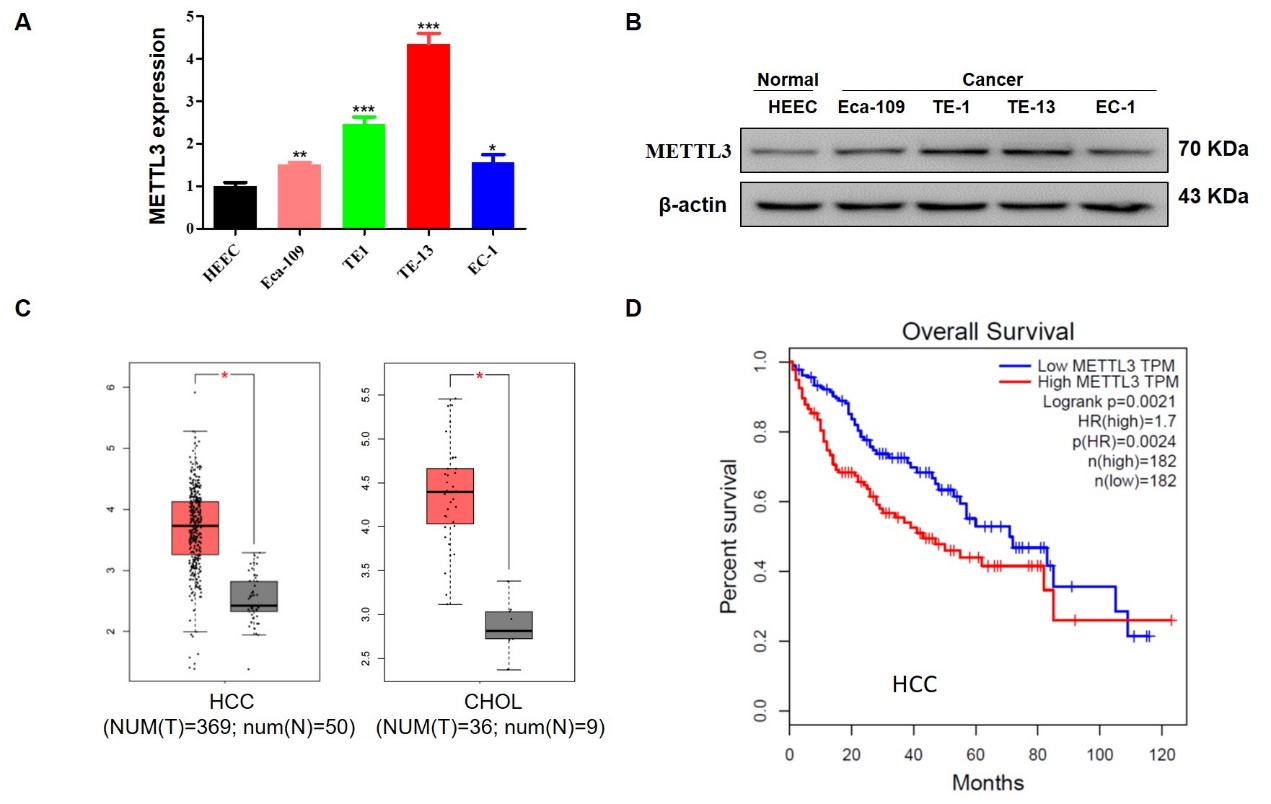


**Supplementary Figure 1.** High expression of METTL3 in cancer indicates the poor prognosis. **(A)** qRT-PCR was used to analyze levels of METTL3 in the indicated ESCC cell lines and normal esophageal epithelial cells. **(B)** Western blotting was used to analyze levels of METTL3 in the indicated ESCC cell lines and normal esophageal epithelial cells. **(C)** Results based on the TCGA (http://software.broadinstitute.org/software/igv/tcga) databases showed the higher expression level of METTL3 in HCC and cholangiocarcinoma. **(D)** Kaplan-Meier and log-rank testing showed the prognosis of patients in the HCC cohort. Data are presented as mean ± SD. *P < 0.05, **P < 0.01, ***P < 0.001.


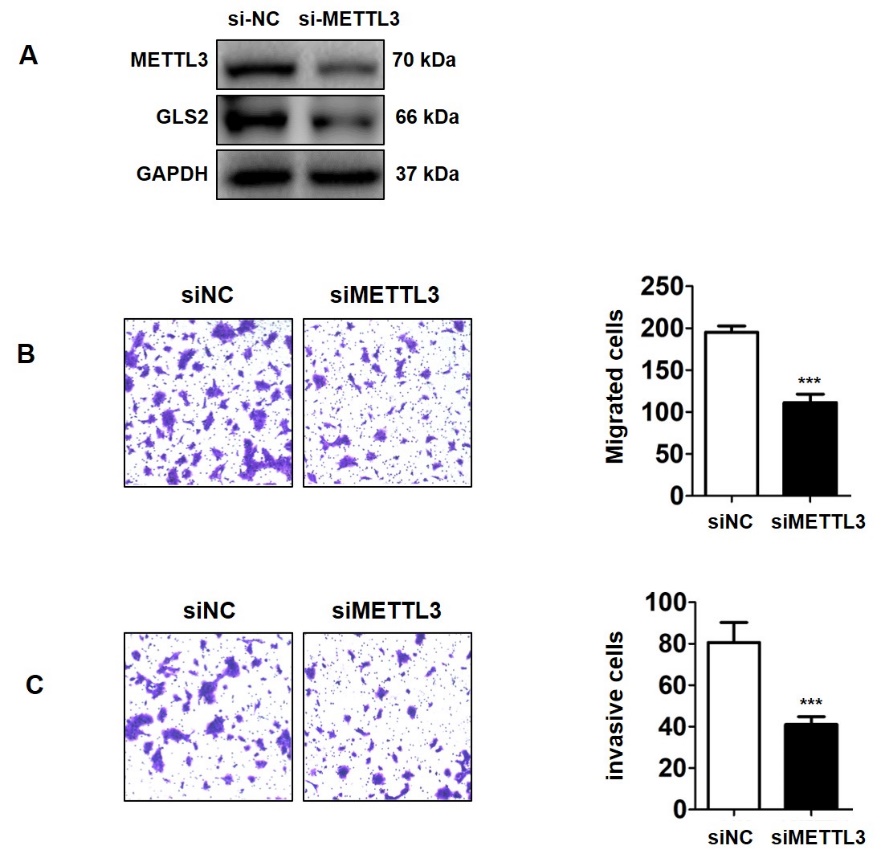


**Supplementary Figure 2.** Downregulation of METTL3 expression inhibits the migration and invasion of TE13 cells. **(A)** Western blot analyses of the expression of METTL3 extracts from TE13 cells transfected with siMETTL3 or siNC. **(B)** Transwell migration assays were used to estimate the effects of downregulation of METTL3 on TE13 cells migration. **(C)** Transwell invasion assays were used to estimate the effects of downregulation of METTL3 expression on TE13 cells invasion. (The quantitative data are presented in the histograms and were assessed with a two-tailed unpaired Student's *t* test. Data are presented as the mean ± SD. ****P* < 0.001.)


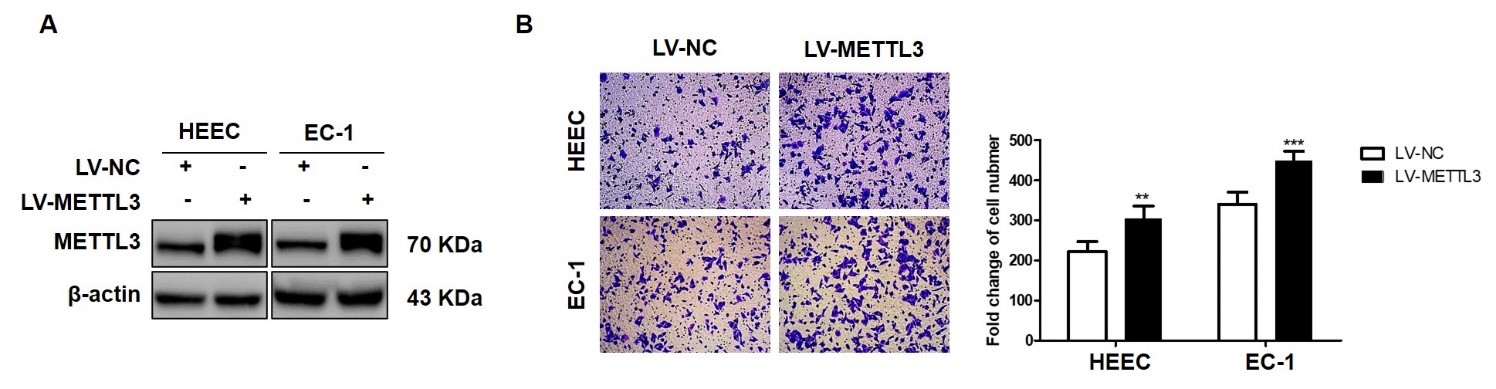


**Supplementary Figure 3.** Overexpression of METTL3 promotes HEEC and EC-1 cells migration. **(A)** Western blot analysis of the expression of METTL3 in cells infected with pLenti-METTL3 or pLenti-vector. **(B)** Transwell migration assays revealed that overexpression of METTL3 increased the migration ability of HEEC and EC-1 cells. Data are presented as mean ± SD. **P* < 0.05, ***P* < 0.01, ****P* < 0.001.


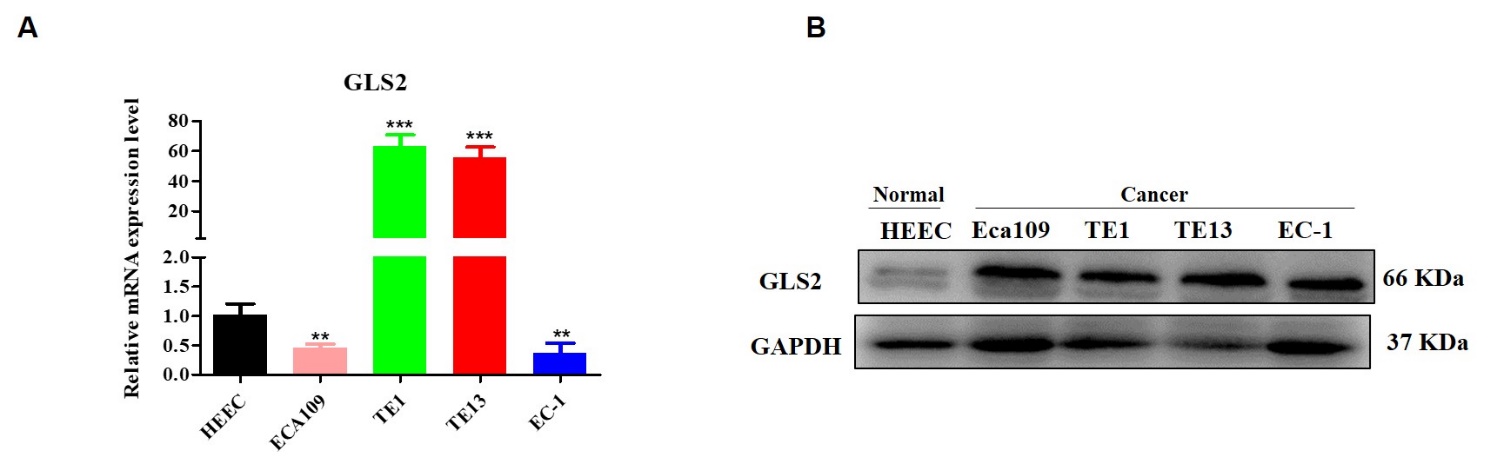


**Supplementary Figure 4.** GLS2 expression is higher in ESCC cell lines compared with HEEC cells. **(A)** qRT-PCR was used to analyze levels of GLS2 in the indicated ESCC cell lines and normal esophageal epithelial cells. **(B)** Western blotting was used to analyze levels of GLS2 in the indicated ESCC cell lines and normal esophageal epithelial cells. Data are presented as mean ± SD. *P < 0.05, **P < 0.01, ***P < 0.001.

## Supplementary Tables

**Supplementary Table 1** Patient demographic and clinical characteristics of 101 ESCC patients

| **Characteristics** | **of cases** |
| --- | --- |
| **Age (years)** |  |
| <65 | 50 (49.50%) |
| ≥65 | 51 (50.50%) |
| **Gender** |  |
| Male | 84 (83.17%) |
| Female | 17 (16.83%) |
| **Primary tumor (pT)** |  |
| T1 | 3 (2.97%) |
| T2 | 19 (18.81%) |
| T3 | 70 (69.31%) |
| T4 | 2 (1.98%) |
| Missing data | 7 (6.93%) |
| **Regional lymph node (pN)** |  |
| N0 | 51 (50.50%) |
| N1 | 30 (29.70%) |
| N2 | 16 (15.84%) |
| N3 | 4 (3.96%) |
| **Distant Metastasis (pM)** |  |
| M0 | 101 (100%) |
| M1 | 0 (0.00%) |
| **TNM stage** |  |
| I | 5 (4.95%) |
| II | 44 (43.56%) |
| III | 39 (38.61%) |
| IV | 6 (5.94%) |
| Missing data | 7 (6.93%) |

**Supplementary Table 2** 26 candidate genes

|  |  | **m6A regulation INFO** | | **gene regulation INFO** | |
| --- | --- | --- | --- | --- | --- |
| **geneName** | **peak_annotation** | **log_2_(FC)** | **pval** | **log_2_(FC)** | **pval** |
| GLS2 | 3' UTR | -0.82 | 0 | -1.32 | 0 |
| PTPRZ1 | 3' UTR | -0.65 | 0.01 | -1.36 | 0 |
| HIST2H4A | 5' UTR | -2.96 | 0 | -1.28 | 0 |
| FTO | Exon | -0.64 | 0.02 | -1.09 | 0 |
| HIST1H2BJ | 3' UTR | -0.61 | 0.02 | -1.08 | 0.01 |
| DHRS12 | 3' UTR | -2.66 | 0 | 1.02 | 0.04 |
| MDGA1 | 3' UTR | -1.17 | 0.01 | 1.03 | 0.02 |
| MYBL1 | Exon | -0.83 | 0.05 | 1.09 | 0 |
| NPIPB3 | 3' UTR | -2.02 | 0 | 1.12 | 0.02 |
| ZNF713 | 3' UTR | -0.8 | 0.02 | 1.12 | 0.03 |
| RASEF | 5' UTR | -0.65 | 0.03 | 1.12 | 0 |
| CALD1 | 3' UTR | -0.51 | 0 | 1.19 | 0 |
| NUS1P1 | Exon | -1.11 | 0 | 1.22 | 0.02 |
| WIPF1 | 5' UTR | -0.62 | 0.05 | 1.22 | 0 |
| LIPA | 5' UTR | -2.54 | 0.05 | 1.26 | 0 |
| ADGRL2 | 3' UTR | -0.9 | 0.02 | 1.26 | 0.02 |
| TSPAN3 | 3' UTR | -0.66 | 0 | 1.41 | 0 |
| KIAA1549L | 3' UTR | -0.56 | 0.02 | 1.43 | 0 |
| TEAD1 | 3' UTR | -1.2 | 0 | 1.45 | 0 |
| C2CD4C | 3' UTR | -0.99 | 0.02 | 1.48 | 0.02 |
| FHOD3 | Exon | -1.05 | 0.02 | 1.77 | 0 |
| AL135905 | 5' UTR | -0.7 | 0.01 | 1.98 | 0 |
| ZNF260 | 3' UTR | -1.02 | 0.01 | 2.1 | 0 |
| VIM | 3' UTR | -2.2 | 0.01 | 2.34 | 0 |
| ROR1 | 3' UTR | -3.25 | 0.01 | 2.4 | 0 |
| CBSL | Exon | -2.68 | 0 | 3.31 | 0.01 |
